# The *Ascosphaera apis* Invasion of *Apis cerana* Worker Larvae: Long Non-Coding RNA-Mediated Regulation

**DOI:** 10.3390/biology15100793

**Published:** 2026-05-15

**Authors:** Yunzhen Yang, Kaiyao Zhang, Genchao Gan, Shuai Zhou, Qingwei Tan, Jianfeng Qiu, Dafu Chen, Zhongmin Fu, Rui Guo

**Affiliations:** 1 College of Bee Science, Fujian Agriculture and Forestry University, Fuzhou 350002, China; yangyunzhen0120@163.com (Y.Y.); kaiyao1223@126.com (K.Z.); gangenchao2025@163.com (G.G.); 15837656300@163.com (S.Z.); tanqingwei@fafu.edu.cn (Q.T.); jfqiu@fafu.edu.cn (J.Q.); dfchen826@fafu.edu.cn (D.C.); 2 National and Local United Engineering Laboratory of Natural Biotoxin, Fuzhou 350002, China; 3 Apitherapy Research Institute of Fujian Agriculture and Forestry University, Fuzhou 350002, China

**Keywords:** *Ascosphaera apis*, non-coding RNA, long non-coding RNA, miRNA, honeybee, *Apis cerana*

## Abstract

*Ascosphaera apis*, an obligate lethal fungal pathogen that infects bee larvae, and causes chalkbrood disease, poses a significant threat to the global beekeeping industry. Long non-coding RNAs (lncRNAs) are employed by pathogens to enhance infectivity and evade host immunity. Here, lncRNAs in *A. apis* spores (AaCK group) and the guts of 4-, 5-, and 6-day-old *Apis cerana cerana* worker larvae inoculated with *A. apis* spores (AaT1, AaT2, and AaT3 groups) were identified, characterized, and validated. Additionally, the expression pattern of fungal lncRNAs during infection was analyzed, followed by an investigation of the regulatory manners and roles of differentially expressed lncRNAs (DElncRNAs). A total of 1379 lncRNAs were identified in AaCK, AaT1, AaT2, and AaT3 groups using bioinformatics, involving various types such as sense lncRNAs, antisense lncRNAs, bidirectional lncRNAs, intergenic lncRNAs, and intronic lncRNAs. Additionally, 4, 9, and 75 up-regulated lncRNAs as well as 2, 1, and 15 down-regulated ones were identified in the 4-, 5-, and 6-day-old larval guts following *A. apis* inoculation. Fifteen DElncRNAs as potential antisense lncRNAs may interact with 15 sense-strand mRNAs in the AaCK vs. AaT3 comparison group. *Cis*-acting analysis identified 10, 16, and 136 upstream and downstream genes of DElncRNAs in the aforementioned comparison groups, involving a series of GO terms and KEGG pathways like metabolic process and biosynthesis of secondary metabolites. Following the *trans*-acting investigation, 752, 821, and 1327 co-transcribed genes with DElncRNAs were discovered, spanning an array of functional terms and pathways such as biological processes and glycerophospholipid metabolism. Analysis of a competing endogenous RNA (ceRNA) network indicated that 1 and 5 DElncRNAs in the AaCK vs. AaT1 and AaCK vs. AaT3 comparison groups potentially targeted 1 and 2 miRNAs, further targeting 208 and 286 mRNAs, respectively. Further analysis identified one ceRNA axis relevant to the MAPK signaling pathway and several ceRNA networks associated with the biosynthesis of secondary metabolites. Finally, RT-qPCR results confirmed that the expression trends of six randomly selected DElncRNAs were consistent with those in the transcriptome data. These findings not only offer a foundation for elucidating the mechanisms underlying DElncRNA-mediated *A. apis* infection but also enrich our understanding of honeybee host–fungal pathogen interactions.

## Simple Summary

Chalkbrood disease, caused by the lethal fungal pathogen *Ascosphaera apis*, poses a severe threat to honeybees and the global beekeeping industry. Pathogens often utilize specific molecules like long non-coding RNAs (lncRNAs) to boost their infectivity and bypass host defenses. Here, we performed an in-depth investigation of the lncRNAs produced by *A. apis* invading the Asian honeybee (*Apis cerana*) worker larvae. We identified 1379 fungal lncRNAs and observed significant changes in their expression across different stages of infection. Comprehensive analyses revealed that these lncRNAs may act as critical regulators to promote fungal growth and disease progression via a diverse array of regulatory mechanisms, including antisense lncRNA-mediated modulation, *cis*- and *trans*-acting regulatory effects, and competing endogenous RNA (ceRNA) network activity. Our data elucidate whether and how lncRNAs modulate the *A. apis* infection of *A. cerana* worker larvae, laying a basis for clarifying the underlying mechanisms.

## 1. Introduction

Long non-coding RNAs (lncRNAs) are a class of RNA molecules longer than 200 nt that do not encode proteins or only translate into short peptides. They are generally transcribed by RNA polymerase II and possess similar structural features to mRNAs, including a 5′-cap structure and a 3′-poly(A) tail [1]. They mainly regulate the expression of neighboring or distant genes through multiple manners, including epigenetics, *cis*-regulation, and *trans*-acting regulation [2], thereby extensively participating in various biological processes such as ontogenesis, metabolic regulation, immune response, and disease occurrence and development [3]. Some lncRNAs can also serve as precursors of microRNAs (miRNAs) to participate in gene expression regulation [4].

In the last decade, the functions of lncRNAs in the interactions between pathogens (e.g., viruses, bacteria, and parasites) and hosts have been extensively studied [5,6,7,8]. Pathogens can express their own lncRNAs to enhance infectivity and evade host immune responses [9,10,11]. For instance, polyadenylated nuclear RNA (PAN RNA), a multifunctional lncRNA encoded by Kaposi’s sarcoma-associated herpesvirus (KSHV), impairs the immunomodulatory function of human cells by interfering with the ability of transcription factors IRF4/PU.1 to activate the interleukin-4 (IL-4) promoter [9]. In *Staphylococcus aureus*, lncRNAs SSR42 and RNAIII act as effector molecules of the repressor of surface proteins (Rsp), controlling the cytotoxicity of bacteria by modulating the transcription and translation of the virulence factor α-toxin [10]. The var antisense lncRNA produced by *Plasmodium falciparum* (the human malaria parasite) exerts an activating effect during the transcription of var genes, thereby enhancing the virulence of the pathogen [11]. Li et al. [12] reported that during infection of hosts by *Cryptosporidium parvum*, the parasite delivers several of its putative lncRNAs (e.g., Cdg7_FLc_0990 and Cdg7_FLc_1000) into host cells, enabling the pathogen to survive and establish infection within host cells by inhibiting the transcription and expression of host genes [12].

However, compared with the aforementioned pathogens, studies on the roles of fungal lncRNAs during infection is still in its infancy, especially in the field of honeybee fungal pathogens. Amidst the global decline of pollinators, honeybee health has emerged as a major scientific concern. Throughout their life cycle, honeybees are continuously challenged by a myriad of biotic and abiotic stressors, including ectoparasitic mites, microbial pathogens, and natural predators [13]. Among these diverse biological threats, *Ascosphaera apis*, an obligate lethal fungal pathogen, is particularly devastating. It is highly infectious to both the worker and drone larvae of *Apis mellifera* and *Apis cerana*. The chalkbrood disease caused by *A. apis* infection poses a significant threat to the global beekeeping industry [14]. Honeybees become infected by ingesting *A. apis* spores, which germinate in the larval gut, penetrate the intestinal wall, proliferate throughout the hemocoel, and ultimately rupture the body surface to form chalkbrood mummies [15]. Currently, control measures for chalkbrood disease are extremely limited, and existing chemical interventions are ineffective and fail to fundamentally prevent disease prevalence. Therefore, in-depth elucidation of the molecular pathogenic mechanisms of *A. apis* is an urgent prerequisite for developing novel and efficient control strategies. In a previous work, we identified 379 lncRNAs in *A. apis* based on deep sequencing and bioinformatics [16], providing a basis for further investigation. However, whether and how lncRNAs regulate the *A. apis* infection of bee larvae is currently largely unknown.

The gut is not merely a passive infection target but a dynamic ecological micro-environment actively defended by resident microbiota against fungal invaders [17]. Based on the pathogenesis and life cycle of *A. apis*, we selected fungal spores and the infected midguts of 4-, 5-, and 6-day-old *A. cerana* worker larvae to investigate this interaction. To address this gap, transcriptome sequencing of the *A. cerana* worker larval gut samples and *A. apis* spore samples were conducted using strand-specific cDNA library-based RNA-seq technology, followed by comprehensive analyses of regulatory manners and roles of differentially expressed lncRNAs (DElncRNAs). Findings from the present study could offer novel insights into the pathogenic mechanism of *A. apis* from the perspective of lncRNA regulation, thereby laying a foundation for elucidating the molecular basis of *A. apis*-*A. cerana* interaction. Additionally, our data provide a valuable reference for the future development of lncRNA-based diagnostic and therapeutic strategies against chalkbrood disease.

## 2. Materials and Methods

### 2.1. Biological Materials

*A. apis* was previously isolated from chalkbrood mummies and deposited at the China General Microbiological Culture Collection Center (No. 40895) [16]. *A. cerana* worker larvae were obtained from three colonies maintained at the teaching apiary of the College of Bee Science and Biomedicine, Fujian Agriculture and Forestry University (Fuzhou, China).

### 2.2. Spore Purification

The *A. apis* spores were purified according to our previously established methods [18]. Briefly, the preserved strain was inoculated onto potato dextrose agar (PDA) plates and cultured at 33 ± 0.5 °C for 10 days. Ascocarps were collected in a biosafety cabinet, and pure spores were obtained after tissue grinding and differential centrifugation. The spore concentration was determined using a hemocytometer, and the purified spores were stored at 4 °C for subsequent use.

### 2.3. Larval Inoculation and Sample Collection

*A. cerana* worker larvae were inoculated and gut samples were prepared following the protocol described by Guo et al. [19] with minor modifications. Briefly, 2-day-old worker larvae (*n* = 48) were obtained from experimental colonies with no clinical signs of chalkbrood. The larvae were transferred to 6-well plates for pre-culture, and maintained in a constant-temperature incubator under standard artificial rearing conditions (35 °C, 95% relative humidity, RH). When larvae reached three days old, each larva was individually transferred to a single well of a 48-well plate (one larva per well), and inoculated with artificial diet containing *A. apis* spores at a final concentration of 1 × 10^7^ spores/mL.

Gut tissues were respectively dissected from 4-, 5-, and 6-day-old larvae, with the resulting treatment group designated as AaT1, AaT2, and AaT3, respectively. Purified spores served as the control group (AaCK). A total of three independent biological replicates were set for each treatment group and the control group. For each biological replicate, three gut samples were pooled, snap-frozen in liquid nitrogen, and stored at −80 °C for subsequent analysis. All procedures were conducted in triplicate to ensure reproducibility.

### 2.4. Library Construction and Sequencing

Total RNA was extracted from AaCK, AaT1, AaT2, and AaT3 groups and rRNA-depleted to enrich for mRNA and ncRNA. Strand-specific libraries were constructed using the dUTP method: enriched RNA was fragmented, followed by first-strand synthesis with random hexamers and second-strand synthesis incorporating dUTP. After end-repair, A-tailing, and adapter ligation, the dUTP-marked strands were digested with Uracil-N-Glycosylase (UNG). Libraries were size-selected, PCR-amplified, and sequenced on the Illumina HiSeq™ 4000 platform by Gene Denovo Biotechnology Co., Ltd. (Guangzhou, China).

### 2.5. Data Processing and Quality Control

Raw reads were processed using fastp to obtain high-quality clean data based on the following filtering criteria: (1) removal of adapter-contaminated reads, (2) removal of reads with more than 10% ambiguous bases (N), (3) removal of poly-A sequences; and (4) removal of low-quality reads (Q ≤ 20 for >50% of bases). After filtering, the proportions of retained clean reads in the three treatment groups and the control group were 76,593,924 (99.90%), 90,870,608 (99.91%), 83,339,288 (99.90%), and 104,621,402 (99.00%), respectively, indicating high-quality sequencing data suitable for further analyses. The resulting clean reads were first mapped to the *A. cerana* reference genome (GCF_029169275.1 (AcerK_1.0)). Unmapped reads were then screened against the *A. apis* ribosomal RNA database (https://www.ncbi.nlm.nih.gov/nuccore/M83264.1, accessed on 15 February 2026) by using Bowtie2, and any rRNA-mapped reads were discarded. Finally, the remaining reads were aligned to the *A. apis* reference genome (GCA_001636715.1 (AAP 1.0)) with HISAT2 for subsequent downstream analyses.

### 2.6. LncRNAs Prediction and Structural Characteristic Analysis

Transcripts were reconstructed from clean reads using StringTie (v1.3.4), and those with length ≥ 200 bp and exon number ≥ 2 were retained. The protein-coding potential of the remaining transcripts was predicted utilizing CPC2 and CNCI software (v2), and the intersection of results was considered as reliable candidate lncRNAs. Expression levels of lncRNAs were calculated using the FPKM (fragments per kilobase of transcript per million mapped reads) method. The numbers of five types of lncRNAs, including intergenic lncRNAs, bidirectional lncRNAs, intronic lncRNAs, antisense lncRNAs, and sense overlapping lncRNAs, were counted and compared.

### 2.7. Small RNA-Seq Data Source

Our team has deposited the microRNA sequencing data of *A. apis* in the NCBI Sequence Read Archive (SRA) database under BioProject accession number PRJNA560456 [20].

### 2.8. Analysis of Antisense LncRNAs

Antisense lncRNAs regulate gene silencing, transcription, and mRNA stability by binding to the sense-strand mRNAs [21]. RNAplex software (version 2.6.4) was employed to predict the complementary pairing between antisense lncRNAs and mRNAs. The optimal base-pairing relationship was predicted by calculating the minimum free energy based on thermodynamic structures. The resulting lncRNA–mRNA pairs were obtained, and the predicted target genes were aligned to the GO database (http://geneontology.org/, accessed on 25 February 2026) and KEGG database (http://www.genome.jp/kegg/, accessed on 25 February 2026) for functional and pathway annotations.

### 2.9. Differential Expression Analysis of lncRNAs

The edgeR software (version 4.0.16) was used to perform differential expression analysis of lncRNAs. DElncRNAs were screened with a threshold of *p*-value (FDR-adjusted) < 0.05 and |log_2_ fold change| ≥ 1 (|log_2_FC| ≥ 1). Expression clustering analysis of DElncRNAs from the three comparison groups (AaCK vs. AaT1, AaCK vs. AaT2, and AaCK vs. AaT3) was conducted using the OmicShare platform (www.omicshare.com) with default parameters.

### 2.10. Analysis of Cis-Acting DElncRNAs

Some lncRNAs function in association with their adjacent protein-coding genes. lncRNAs located upstream can regulate gene expression at the transcriptional or post-transcriptional level through targeting promoters or other *cis*-acting elements of co-expressed genes, while lncRNAs located in the 3′ UTR or downstream of genes may participate in other regulatory mechanisms [22]. Protein-coding genes within 10 kb upstream or downstream of lncRNAs were screened as target genes. GO and KEGG database annotations were performed for the target genes of DElncRNAs using the OmicShare platform.

### 2.11. Analysis of Trans-Acting DElncRNAs

Some lncRNAs influence chromatin structure, transcriptional elongation efficiency, or mRNA processing by recruiting or binding specific chromatin-modifying proteins, transcription factors, or RNA-binding proteins (RBPs) [23]. Target genes were predicted by analyzing the expression correlation between lncRNAs and protein-coding genes across samples. The Pearson correlation coefficient method was utilized to evaluate the expression correlation between lncRNAs and protein-coding genes. Protein-coding genes with an absolute correlation coefficient greater than 0.999 were selected for GO and KEGG database annotation. This threshold was selected to minimize false-positive predictions in our moderate-size dataset, to focus on biologically credible trans regulatory pairs without genomic location constraints, and to ensure the reliability of downstream functional enrichment analyses.

### 2.12. Construction and Investigation of ceRNA Regulatory Networks

By using RNAhybrid [24], Miranda [25], and TargetScan [26], target DEmiRNAs of DElncRNAs and DEmiRNA-targeted mRNAs were predicted. On basis of KEGG pathway annotations, target mRNAs associated with pathogen proliferation and infection-related pathways were screened. These candidate mRNAs, along with their upstream regulatory DEmiRNAs and DElncRNAs, were integrated to construct DElncRNA–DEmiRNA and DElncRNA–DEmiRNA–mRNA regulatory networks. All networks were visualized using Cytoscape software (version 3.10.3) [27].

### 2.13. RT-qPCR Validation

To verify the RNA-seq data, two DElncRNAs were randomly selected from each comparison group. Specific primers (Table 1) were designed using Primer Premier 6.0 and synthesized by Sangon Biotech (Shanghai, China). Total RNA was extracted and cDNA synthesis was then performed with the Hifair^®^ III 1st Strand cDNA Synthesis Kit (Yeasen, China) using Random Primers N6. The resultant cDNA was used as templates for RT-qPCR detection. The *A. apis* 5.8 *S rRNA* (GenBank accession number: U68313.1) was used as the internal reference. RT-qPCR was performed following the described protocol by Chen et al. [28]. The 20 μL reaction mixture contained: 10 μL SYBR Green Dye, 1 μL each of forward and reverse primers (4 μmol·L^−1^), 3 μL cDNA template, and DEPC water up to 20 μL; 95 °C for 1 min, followed by 40 cycles of 95 °C for 15 s and 56 °C for 30 s. All experiments were conducted in triplicates. Relative expression levels were calculated using the 2^−ΔΔCt^ method [29], and data were analyzed using GraphPad Prism 10.0.

## 3. Results

### 3.1. Identification and Categorization of LncRNAs in A. apis

**Figure 1:**
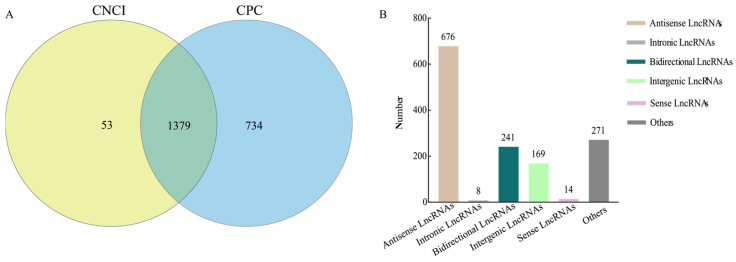
Analysis of lncRNAs in *A. apis*. (**A**) Venn diagram of the intersection of lncRNAs respectively predicted by CNCI and CPC software; (**B**) classification of the identified lncRNAs.

By using CPC and CNCI software, 1432 and 2113 lncRNA were respectively predicted, with 1379 common ones (Figure 1A). As shown in Figure 1B, the identified lncRNAs included 14 sense lncRNAs, 676 antisense lncRNAs, 8 intronic lncRNAs, 241 bidirectional lncRNAs, 169 intergenic lncRNAs, and 271 other lncRNAs.

### 3.2. Expression Profile of A. apis LncRNAs During the Infection Process

**Figure 2:**
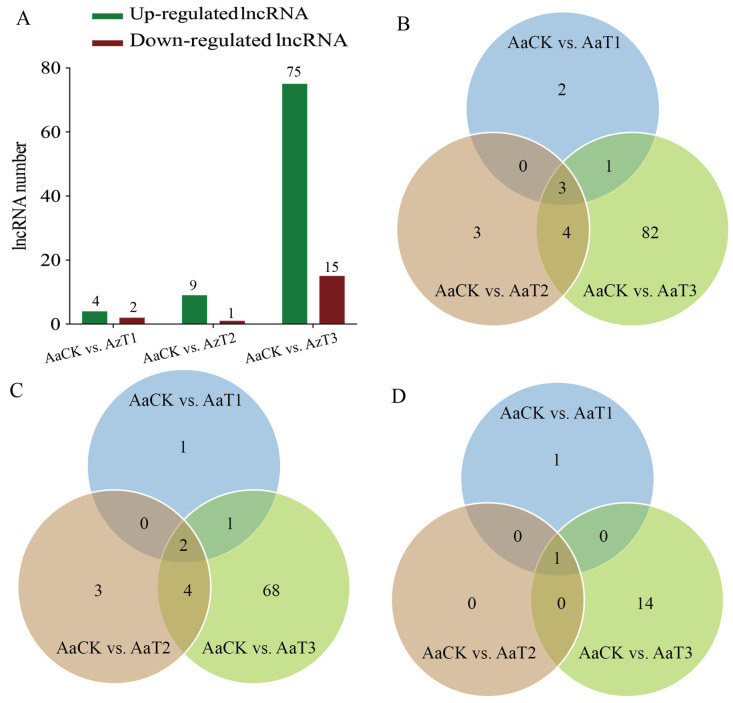
Differential expression analysis of lncRNAs in *A. apis* infecting the *A. cerana* worker larvae. (**A**) Numbers of DElncRNAs identified in AaCK vs. AaT1, AaCK vs. AaT2, and AaCK vs. AaT3; (**B**–**D**) Venn diagrams of DElncRNAs, up-regulated lncRNAs, and down-regulated lncRNAs across three comparison groups.

Differential expression analysis suggested that 4 (2), 9 (1), and 75 (15) up-regulated (down-regulated) lncRNAs were identified in the AaCK vs. AaT1, AaCK vs. AaT2, and AaCK vs. AaT3 comparison groups, respectively (Figure 2). Additionally, two up-regulated lncRNAs (MSTRG.2659.1 and MSTRG.3070.1) and one down-regulated lncRNA (MSTRG.2213.1) were shared by these three comparison groups. The numbers of unique DElncRNAs in each group were 2, 3, and 82, respectively.

### 3.3. Analysis of Antisense DElncRNAs in A. apis During the Infection Process

**Figure 3:**
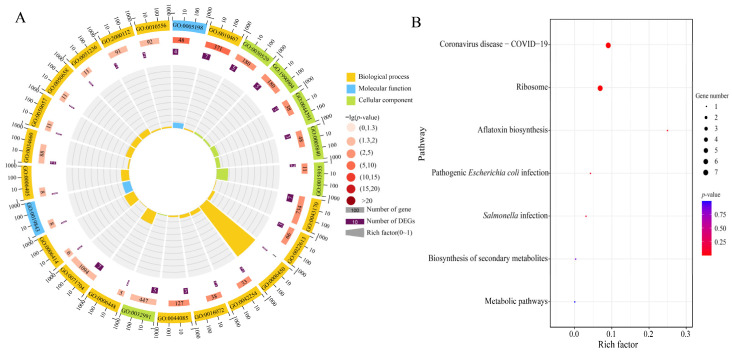
GO terms and KEGG pathways relative to of target mRNAs of antisense DElncRNAs in *A. apis* invading the *A. cerana* worker 6-day-old larva. (**A**) Loop diagram of functional terms associated with target mRNAs; (**B**) pathways associated with target mRNAs.

In the AaCK vs. AaT3 comparison group, 15 DElncRNAs were predicted to target 15 mRNAs. However, no target mRNAs were identified for DElncRNAs in the other two comparison groups. The above-mentioned target mRNAs were involved in 23 cellular component-associated terms (e.g., intracellular part, intracellular, and cell), 95 biological process-related terms (e.g., gene expression macromolecule metabolic process and ribonucleoprotein complex biogenesis), and 15 molecular function terms (e.g., molecular function, including molecular function, and binding) (Figure 3A). In addition, these targets were engaged in five pathways, such as ribosome, aflatoxin biosynthesis, and metabolic pathways (Figure 3B).

### 3.4. Cis-Acting Effect of DElncRNAs in A. apis During the Infection Process

In the AaCK vs. AaT1, AaCK vs. AaT2, and AaCK vs. AaT3 comparison groups, 10, 16, and 136 upstream and downstream genes of DElncRNAs were identified, respectively.

**Figure 4:**
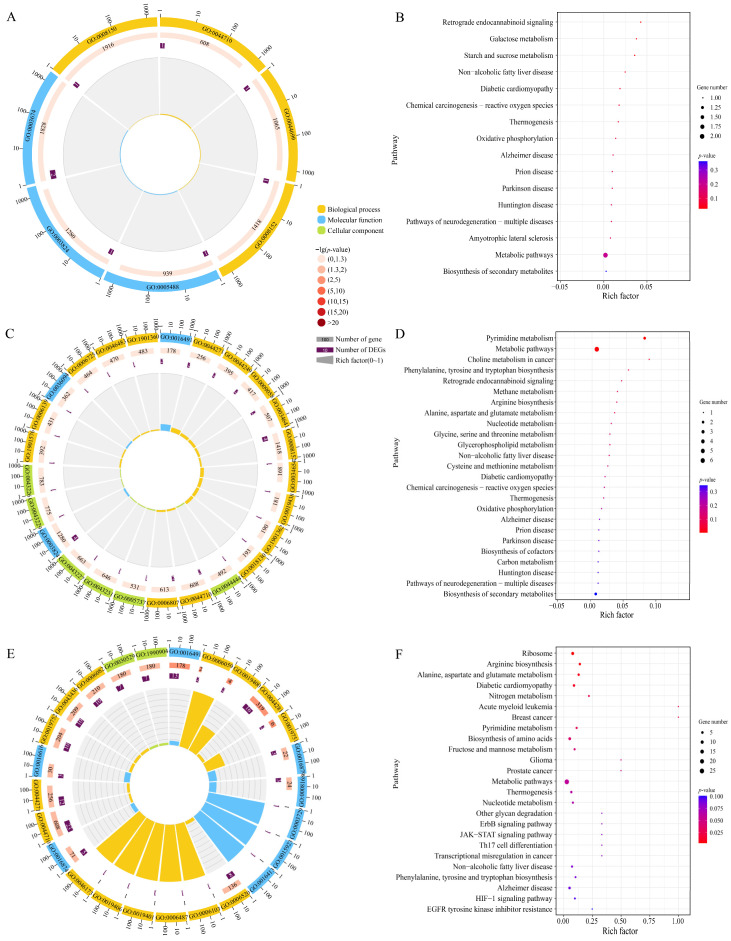
Functional and pathway annotations of upstream and downstream genes of DElncRNAs. (**A**,**C**,**E**) Loop diagrams of GO termed enriched by upstream and downstream gene of DElncRNAs in *A. apis* infecting 4-, 5-, and 6-day-old larvae; (**B**,**D**,**F**) KEGG pathways annotated by upstream and downstream gene of DElncRNAs in *A. apis* infecting 4-, 5-, and 6-day-old larvae.

These upstream and downstream genes were involved in 7, 39, and 399 GO terms, such as molecular function, catalytic activity, and metabolic process (Figure 4A,C,E). They were engaged in 16, 28, and 120 KEGG pathways, including metabolic pathways, biosynthesis of secondary metabolites, and ribosome (Figure 4B,D,F).

### 3.5. Trans-Acting Effect of DElncRNAs in A. apis During the Infection Process

**Figure 5:**
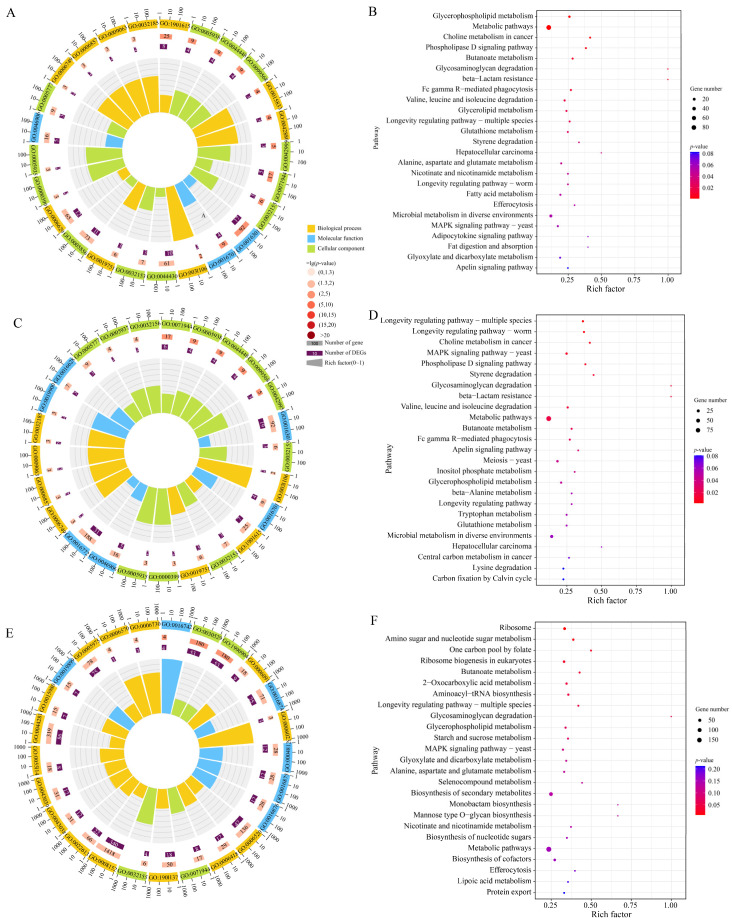
Loop graphs and bubble diagrams (Top 25) of DElncRNAs-targeted mRNAs in the *A. apis* at AaCK vs. AaT1 comparison (**A**,**B**), AaCK vs. AaT2 comparison (**C**,**D**), and AaCK vs. AaT3 comparison (**E**,**F**).

A total of 752, 821, and 1327 co-transcribed genes associated with DElncRNAs were identified in the AaCK vs. AaT1, AaCK vs. AaT2, and AaCK vs. AaT3 comparison groups, respectively. These co-transcribed genes were enriched in 883, 1015, and 1352 GO terms, including molecular function, biological process, and metabolic process (Figure 5A,C,E). Furthermore, they were significantly assigned to 224, 275, and 120 KEGG pathways, such as glycerophospholipid metabolism, metabolic pathways, and ribosome (Figure 5B,D,F).

### 3.6. CeRNA Networks of DElncRNAs in A. apis During the Infection Process

Based on the predicted targeting relationships, regulatory networks among DElncRNAs, miRNAs, and mRNAs were constructed and investigated. The results demonstrated that one DElncRNA in the AaCK vs. AaT1 comparison group targeted one miRNA, which further targeted 208 mRNAs, forming a ceRNA network. In the AaCK vs. AaT3 comparison group, five DElncRNAs were predicted to target two miRNAs, further targeting 286 mRNAs.

**Figure 6:**
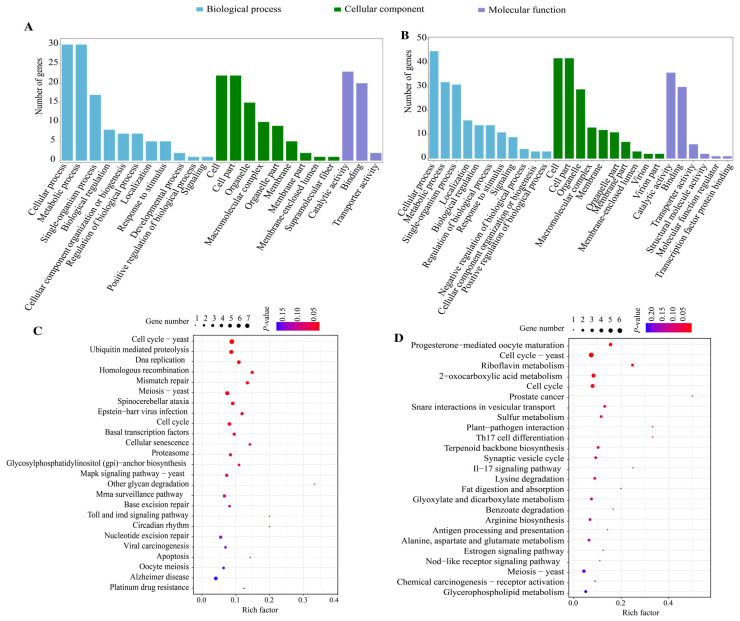
Functional enrichment and structural analysis of the ceRNA regulatory networks. (**A**,**C**) GO terms and KEGG pathways associated with target mRNAs in the AaCK vs. AaT1 ceRNA network; (**B**,**D**) GO terms and KEGG pathways associated with target mRNAs in the AaCK vs. AaT3 ceRNA network.

It was found that the target mRNAs of miR414-x were associated with 23 and 27 GO terms (e.g., cellular process, metabolic process, and cell part) (Figure 6A,B) as well as 25 KEGG pathways (e.g., cell cycle-yeast, ubiquitin mediated proteolysis, meiosis-yeast, and MAPK signaling pathway-yeast) in the AaCK vs. AaT1 and AaCK vs. AaT3 comparison groups, respectively (Figure 6C,D).

**Figure 7:**
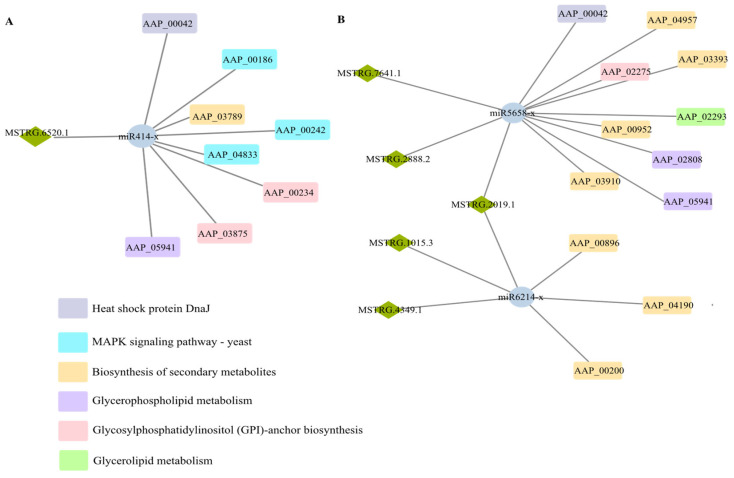
DElncRNA-miRNA-mRNA regulatory networks associated with host infection pathways in AaCK vs. AaT1 (**A**) and AaCK vs. AaT3 (**B**) comparison groups.

Further analyses focusing on fungal proliferation and pathogenesis revealed that only one DElncRNA–DEmiRNA axis was identified in the AaCK vs. AaT1 comparison group, which modulated eight mRNAs associated with the MAPK signaling pathway and glycosylphosphatidylinositol (GPI)-anchor biosynthesis (Figure 7A). In contrast, the regulatory network in the AaCK vs. AaT3 group was more complex, with five DElncRNAs targeting two DEmiRNAs to regulate 11 mRNAs mainly involved in the biosynthesis of secondary metabolites and glycerophospholipid metabolism, suggesting potential stage-related shifts in the lncRNA-mediated regulatory network during *A. apis* infection (Figure 7B).

### 3.7. RT-qPCR Validation of DElncRNAs

**Figure 8:**
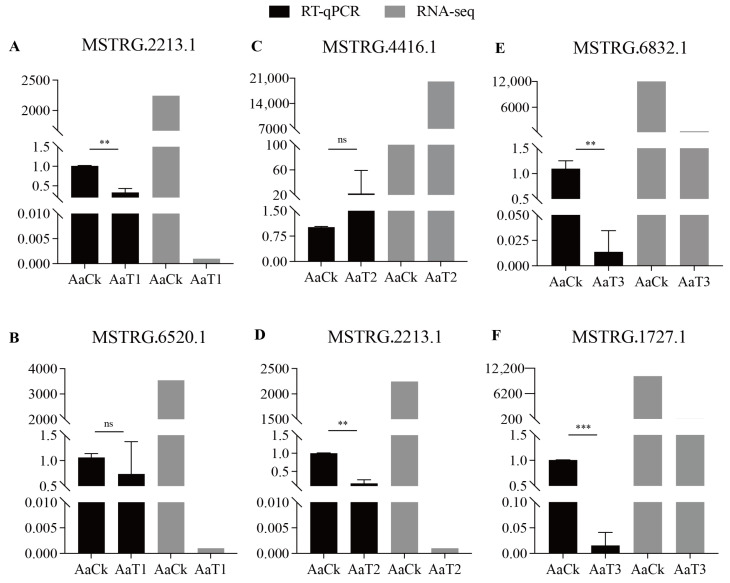
RT-qPCR verification of DElncRNAs. (**A**–**F**) Relative expression levels in AaCK vs. AaT1 (**A**,**B**), AaCK vs. AaT2 (**C**,**D**), and AaCK vs. AaT3 (**E**,**F**) comparison groups. Data are shown as Mean ± SD and subjected to Student’s *t* test (ns: *p* > 0.05, **: *p* < 0.01, ***: *p* < 0.001).

RT-qPCR results suggested that the expression trends of the six randomly selected DElncRNAs from the above-mentioned three comparison groups were consistent with the sequencing results (Figure 8), indicating that the transcriptome data used in this study were reliable.

## 4. Discussion

Previously, Guo et al. [16] identified 379 lncRNAs in mycelia and spores of *A. apis*. Here, on the basis of transcriptome data from *A. apis* spores and *A. apis* infecting the larval guts 1379 novel lncRNAs were identified, much more than those lncRNAs discovered in the mixed samples of *A. apis* spores and mycelia. As highlighted by our study, this significant increase is attributed to two main factors. Technically, it is driven by the unprecedented depth of sequencing and optimized bioinformatic prediction criteria employed in this work, which successfully captured a multitude of low-abundance non-coding transcripts. Biologically, this abundance profoundly reflects the specific fungal response during this specific stage of larval infection. Although in vitro fungal spores maintain a dormant state with extremely low metabolic activity, the active infection process within the larval gut environment compels the pathogen to undergo massive transcriptional reprogramming to adapt to the intricate host–pathogen interactions [30,31]. To rapidly orchestrate the complex interactions of biological macromolecules required to respond to environmental stress, to regulate virulence factors, to remodel cellular structures, and to evade host immunity upon germination, spores likely pre-package these diverse lncRNAs as essential regulatory reserves [32]. The identified lncRNAs in this work expand the *A. apis* lncRNA repertoire, offering valuable resources for further investigation. Following differential expression analysis, 4, 9, and 75 up-regulated DElncRNAs, alongside 2, 1, and 15 down-regulated ones, were screened from the AaCK vs. AaT1, AaCK vs. AaT2, and AaCK vs. AaT3 comparisons, respectively (Figure 2A). We found that the expression level of lncRNAs in *A. apis* increased significantly with the extension of infection time. This is indicative of the alteration of the expression pattern of lncRNAs in *A. apis* during the infection process, suggesting the involvement of DElncRNAs in regulating fungal invasion.

Antisense lncRNAs, transcribed from the complementary strands of genes, are pivotal modulators spanning from chromatin modification to translation [33]. Our study identified 15 antisense DElncRNAs specifically in the *A. apis* infecting the 6-day-old larval gut, which potentially targeted 15 mRNAs enriched in a subseries of functional terms (e.g., methylation, posttranscriptional regulation of gene expression, and nucleic acid binding) and pathways like biosynthesis of secondary metabolites, metabolic pathways, and ribosome (Figure 3A,B). The results demonstrate that *A. apis*-derived antisense lncRNAs may specifically control fungal pathogenicity during the late stage of infection. However, additional work is needed to verify the prediction.

*Cis*-acting lncRNAs regulate the expression of adjacent genes on the same chromosome [34,35]. In the present study, we identified 10, 16, and 136 upstream or downstream genes of DElncRNAs in the 4-, 5-, and 6-day-old larval guts infected by *A. apis*, respectively. Subsequent functional annotation showed that these upstream or downstream genes were annotated to 0, 4, and 47 virulence-related functional terms (transferase activity, hydrolase activity, acting on glycosyl bonds, and mannosidase activity, etc.), respectively. Notably, the progression of the infection was accompanied by a continuous increase in the number of functional terms relative to pathogen proliferation. These results suggested that DElncRNAs extensively regulate *A. apis* proliferation and ribosome metabolism through a *cis*-acting manner.

The *trans*-acting lncRNAs can regulate distant genes across various chromosomes, playing a critical part in growth, development, metabolism, and immune response [36]. In this current work, 752, 821, and 1327 co-transcribed genes with DElncRNAs were detected in three comparison groups. In early infection, the majority of co-transcribed genes were annotated to 62 pathways associated with host invasion and barrier degradation, including O-glycoside hydrolase activity, external structure organization, and filamentous growth. The upregulation of related enzyme-encoding genes enables the *A. apis* to degrade host barriers for invasion and colonization, consistent with the regulatory mechanism of cell wall-degrading enzymes (CWDEs) in various pathogenic fungi [37,38]. As infection progressed, more targets were annotated to 109 that were related to oxidative stress response and glutathione metabolism, indicating that the fungal pathogen was likely to activate endogenous antioxidant systems to neutralize host ROS bursts, thereby achieving immune evasion and persistent infection [39,40].

LncRNAs can indirectly regulate the expression of downstream target genes by competitively binding to miRNAs via the ceRNA mechanism [41]. Host infection by pathogenic fungi relies on cell wall remodeling and infection structure formation for early invasion, and is conservatively regulated throughout the infection process by the MAPK signaling pathway and protein phosphorylation modification [42,43]. Here, it is detected that one DElncRNA in the AaCK vs. AaT1 comparison group could target one DEmiRNA, subsequently regulating five mRNAs engaged in 14 core pathways, such as MAPK signaling pathway, cell cycle, and glycosylphosphatidylinositol (GPI)-anchor biosynthesis. It is hypothesized that during the early stages of infection, DElncRNAs act via ceRNA networks to modulate the MAPK signaling pathway, enabling the pathogen to sense external environmental cues and initiate early colonization [44]. In addition, five DElncRNAs in the AaCK vs. AaT3 comparison group could target two DEmiRNAs, further regulating 286 mRNAs involved in 32 vital pathways like cell cycle-yeast, DNA replication, glycosylphosphatidylinositol (GPI)-anchor biosynthesis, and biosynthesis of secondary metabolites. As the infection progresses, the *A. apis* successfully establishes colonization and undergoes massive proliferation [14]. Consequently, *A. apis* is speculated to shift its pathogenic strategy: it is likely to leverage DElncRNAs to precisely regulate the biosynthesis of secondary metabolites to dismantle host cellular structures and subvert the host immune defense system within the ceRNA network [45]. These findings indicate that lncRNA-mediated ceRNA networks play potential regulatory roles in the whole infection process via stage-specific precise regulation. It should be noted that the proposed DElncRNA-miRNA-mRNA ceRNA axes are computationally predicted using RNAhybrid, Miranda, and TargetScan software. These algorithms primarily rely on sequence complementarity and thermodynamic stability, which cannot fully capture the complexity of in vivo spatial-temporal co-expression and 3D RNA conformations. Therefore, the precise physical interactions require future in vitro validations, ideally via dual-luciferase reporter assays. Currently, fungal ceRNA research predominantly relies on predictions [46]. Although experimentally confirmed fungal models are still lacking, our predicted network topologies closely align with conserved ceRNA paradigms well-established in other eukaryotes [47,48]. While these axes are presented as putative networks, they provide biologically plausible and prioritized candidates for our future functional explorations.

Although the reliability of our sequencing data was confirmed by the RT-qPCR validation of six DElncRNAs, it is important to note that the current functional annotations rely primarily on bioinformatic predictions. A major limitation of this study is the lack of direct in vivo functional assays. Elucidating the precise biological roles of these differentially expressed lncRNAs through functional experiments will be the primary focus of our future research.

## 5. Conclusions

The dynamic change in lncRNA expression profile is accompanied by the infection of *A. cerana* worker larvae with *A. apis*. DElncRNAs are potentially engaged in the proliferation and invasion of *A. apis* through diverse manners, such as antisense transcript pairing, *cis*-/*trans*-regulatory modes, and ceRNA networks.

## Figures and Tables

**Table 1:** Sequences of primers qPCR detection.

Gene	Sequence (5′-3′)
*5.8s* *r* *RNA*	F: GGCACTTTGTTAGGCTTTG
	R: GTTTGGTGACTCTTCTTCCTTC
*MSTRG.4416.1*	F: CGCTCTGTCAACCAGTATGTC
	R: GATGCTTCCGACTCCTCTGA
*MSTRG.2213.1*	F: TGGTTCGTGGAGCGTGATGAT
	R: GCCACTTGTCCGATGAGAGGTA
*MSTRG.6520.1*	F: AGATGTCTGCACTGCCAACCT
	R: CATCATCGCCGTCATCGTCTTG
*MSTRG.1727.1*	F: TGTCCAGTATGCCCTAAAGTGA
	R: CAGCCGACCGAAGTCAAGA
*MSTRG.6832.1*	F: CACAGTCACATCCTCACTCAAC
	R: CGGATTTGCGGATACACACG

## Data Availability

The raw data supporting the conclusions of this article will be made available by the corresponding author on reasonable request.
